# Retraction Note: The effect of early oral stimulation with breast milk on the feeding behavior of infants after congenital cardiac surgery

**DOI:** 10.1186/s13019-023-02408-w

**Published:** 2023-10-14

**Authors:** Xian-Rong Yu, Shu-Ting Huang, Ning Xu, Li-Wen Wang, Zeng-Chun Wang, Hua Cao, Qiang Chen

**Affiliations:** 1 Department of Cardiac Surgery, Fujian Maternity and Child Health Hospital, Affiliated Hospital of Fujian Medical University, Fuzhou, China; 2 Fujian Key Laboratory of Women and Children’s Critical Diseases Research, Fujian Maternity and Child Health Hospital, Fuzhou, China; 3 Department of Cardiovascular Surgery, Union Hospital, Fujian Medical University, Fuzhou, China

**Retraction Note: Journal of Cardiothoracic Surgery (2020) 15:309** **https://doi.org/10.1186/s13019-020-01355-0**

The Editor-in-Chief has retracted this article due to the lack of adherence to the journal's ethical standards and editorial policy. After publication, concerns were raised regarding the trial registration and ethics approval of this study. The authors have provided the ethics approval, which was dated after the patient recruitment start date.

All authors agree to this retraction.

